# Molecular subgroup of periodontitis revealed by integrated analysis of the microbiome and metabolome in a cross-sectional observational study

**DOI:** 10.1080/20002297.2021.1902707

**Published:** 2021-03-25

**Authors:** Hee Sam Na, Suhkmann Kim, Seonghye Kim, Yeuni Yu, Si Yeong Kim, Hyun-Joo Kim, Ju Youn Lee, Jae-Hyung Lee, Jin Chung

**Affiliations:** a Department of Oral Microbiology, School of Dentistry, Pusan National University, Busan, South Korea; b Oral Genomics Research Center, Pusan National University, Busan, South Korea; c Department of Chemistry, Center for Proteome Biophysics, and Chemistry Institute for Functional Materials, Pusan National University, Busan, South Korea; d Interdisplinary Program of Genomic Science, Pusan National University, Busan, South Korea; e Department of Periodontology, School of Dentistry, Pusan National University, Busan, South Korea; f Dental Research Institute, School of Dentistry, Pusan National University, Busan, South Korea; g Department of Oral Microbiology, School of Dentistry, Kyung Hee University, Seoul, South Korea; h Department of Life and Nanopharmaceutical Sciences, Kyung Hee University, Seoul, South Korea; i Kyung Hee Medical Science Research Institute, Kyung Hee University, Seoul, South Korea

**Keywords:** Periodontitis, oral microbiome, saliva, metabolome, multi-omics

## Abstract

**Background**: Periodontitis (PT) is a multifactorial, chronic inflammatory disease that can have heterogeneous clinical presentations. The oral microbiome and its metabolites have been implicated as the causes and regulators of PT pathogenesis. In this study, we assessed the oral microbiome and its metabolome in PT patients to clarify the interactions between the microbiome and its metabolites.**Methods**: A total of 112 subjects were recruited. Buccal and supragingival samples were collected for microbiome analysis. Saliva samples were collected for metabolomic analyses. Microbiome and metabolome data were analyzed and further integrated for combined analysis using various bioinformatics approaches.**Results**: Oral metabolomic analysis identified 28 metabolites distinguishing the healthy (H) and PT groups. PT group were further clustered into two subgroups (PT_G1 and PT_G2) depending on metabolite profiles. Oral microbiome analysis revealed discriminatory bacterial species in the H, PT_G1, and PT_G2 microbiota. Interestingly, PT_G2 had significantly higher concentration of short chain fatty acids and higher abundance of pathogenic bacteria. Integrated analysis of the microbiome and metabolome showed close association.**Conclusion**: Our results provide evidence of a close interplay between the oral microbiome and metabolome. Multi-omics approach including microbiome and microbe-associated metabolites may serve as diagnostic biomarkers and enhance treatment prediction in periodontal disease.

## Introduction

Periodontitis (PT) is a multifactorial and chronic inflammatory disease that leads to the loss of periodontal attachment for the root surface and alveolar bone resorption. PT has heterogeneous clinical presentations, and its diagnosis is primarily based on clinical examination and radiographic parameters [1,2]. Gradually, the traditional culture-based, biochemical, and immunological assays for clinical diagnostics are being replaced by more advanced diagnostic tools [3,4]. Metagenomics focuses on the microbial communities that colonize areas of the human body, such as the oral cavity [5]. PT is initiated by the colonization of periodontal pathogens including Gram-negative *Porphyromonas gingivalis, Treponema denticola*, and *Tannerella forsythia*, which are frequently isolated from subgingival dental plaques in PT patients. These bacteria were initially considered specific pathogens of periodontal disease [6]. A strong correlation between oral bacteria and periodontal disease has been reported [7–11].

Oral microbes are capable of converting the complex chemicals present in saliva into a milieu of metabolites [12]. Recent studies suggest that microbial metabolites are important in connecting the oral microbiota to PT [13,14]. Thus, disruption of the microbiome and the balance of metabolites can induce an excessive inflammatory response resulting in tissue destruction in PT. However, the interplay between the oral microbiota and metabolites as well as their roles in PT development have not been effectively addressed.

Metabolomics involve the high-throughput identification and quantification of the whole ensemble of metabolites in a cell, a tissue, body fluids, or ecological systems [15]. Among various metabolomic platforms, nuclear magnetic resonance (NMR) spectroscopy is a quantitative and robust method with a straightforward and simple sample handling [16,17].

In this study, we applied multi-omics analysis of PT that combined microbiome analysis using 16S rRNA sequencing and metabolome analysis using NMR. We first determined metabolic profiles and grouped PT into two subgroups. The microbial composition and their clinical outcomes were further characterized depending on the metabolic profiles. Our study demonstrates how combining multi-omics data can provide deeper understanding of periodontal disease.

## Materials and methods

### Study population and clinical examination

A total of 112 subjects was recruited in the current study. These included 79 patients with PT and 33 subjects defined as healthy. The subjects were recruited at the Department of Periodontics of Pusan National University Dental Hospital, Yangsan, South Korea. In general, we enrolled individuals who were not pregnant or breastfeeding, had no systemic diseases that may affect the periodontal status, and had not received antibiotics in the last 6 months or undergone periodontal therapy (scaling and root planning) in the last 3 months. Exclusion criteria included the use of anti-inflammatory drugs, acute infection (e.g. herpetic gingivostomatitis), chronic mucosal lesion (e.g. pemphigus and pemphigoid) of the oral cavity, and a current status of smoking.

Full-mouth clinical examinations, including probing depth (PD), clinical attachment level (CAL), gingival index (GI), and plaque index (PI), were carried out by one practitioner. Patients with PT had moderate-to-severe periodontal disease, including PD > 5 mm, clinical attachment loss > 3 mm, and radiographic evidence of distinct bone loss. The healthy control (H) group consisted of individuals with clinically healthy gingival tissues (low scores of bleeding on probing in <10% of the sites and no sites with PD > 3 mm or CAL). For clinical follow up, the clinical examinations were performed again 3 to 6 months after treatment in the PT group.

The experimental protocol was approved by the Institutional Review Board of Pusan National University (PNUDH-2017-023). Written informed consent was obtained from all participants before the study.

### Microbial sample collection

Buccal and supragingival samples were collected with the full-mouth periodontal examination. All participants were requested to refrain from food and oral hygiene (brushing or flossing the teeth) for 2 h before sampling. Samples were collected after isolating the selected sampling site with cotton rolls and gentle air drying. Buccal swab samples were obtained from the mucosa of both the cheeks with a sterile swab and supragingival plaque samples were collected from the molars of each participant using a sterile Gracey curette. Obtained samples were placed in a sterile 1.5 mL microcentrifuge tube and stored at −80°C for subsequent processing.

### Saliva sample collection

Whole saliva was obtained from all study subjects using Salivette (Sarstedt, Numbrecht, Germany). Following plaque sampling, all subjects were asked to rinse their mouth with distilled water 5 min prior to saliva collection. At the time of saliva collection, each subject placed a cotton ball in their mouth for 5 min. The cotton ball was removed and transferred to a plastic tube. Collected samples were centrifuged and the saliva was stored at −80°C until further analysis.

### 16S rRNA metagenomic sequencing and microbiome analysis

DNA from plaque samples was extracted using the MasterPure Gram Positive DNA purification kit (Lucigen, Middleton, WI) according to the manufacture’s protocol. The final concentration was measured with a NanoDrop ND-1000 spectrophotometer (Thermo Fisher Scientific, Waltham, MA) and stored at −80^◦^C until use. The *16S rRNA* genes were amplified from input genomic DNA using 16S V3-V4 primers. The sequencing library was prepared according to the 16S Metagenomic Sequencing Library protocols (Illumina, San Diego, CA). The quality and quantity of the DNA sequencing library were assessed using the PicoGreen assay kit (Molecular Probes, Eugene, OR). The libraries were sequenced using MiSeq platforms (Illumina). Basic microbiome analyses have been previously performed using the QIIME2 [18] and associated plugins.

### ^1^H nuclear magnetic resonance (NMR) spectral acquisition and analysis of saliva samples

For the NMR measurement, saliva samples of the H group and PT patients were thawed at 4°C and centrifuged at 10,000 rpm for 1 min. The supernatant (450 μL) was collected and mixed with 50 μL of phosphate buffer (pH 7.4) in deuterated water containing 20 mM 3-trimethylsilyl-2,2,3,3-tetradeuteropropionicacid-d_4_ (TSP-d_4_; Sigma-Aldrich, St. Louis, MO). TSP-d_4_ was added as the internal standard for chemical shift and quantitative standard. The prepared sample was vortexed and transferred to a 5 mm NMR tube.

^1^H-NMR spectra were acquired using a 600 MHz NMR spectrometer with a liquid probe (Agilent Technologies, Santa Clara, CA) operating at 25°C. A Carr-Purcell-Meiboom-Gill pulse sequence was used to suppress the water and macromolecular signals with the following acquisition parameters: spectral width 9615.4 Hz, 3 s acquisition time, and 128 scans. The baseline and phase correction of the acquired spectra were manually processed; the TSP-d_4_ peak was referenced as 0 ppm with VnmrJ 4.2 software (Aligent Technologies). The spectral region between 0.5 and 10.0 ppm was binned into regions of 0.05 ppm width, omitting the areas of water (from 4.68 to 4.88 ppm). The binned data were normalized, and the multivariate pattern recognition analysis was performed using SIMCA P^+^ (Umetrics, Umeå, Sweden).

Metabolites were identified and quantified with the Chenomx software 7.1 (Chenomx Inc., Edmonton, AB, Canada) based on the 600 MHz NMR library. The metabolite concentrations were normalized by the sum to reduce sample variation. To confirm the significant metabolites between the H group and PT patients, statistical analysis was performed with Student’s *t*-test and ANOVA.

### Bioinformatics analysis, statistical analyses, and visualization

The mean clinical parameters were compared using the paired Student’s t-test or ANOVA test. NMR data were converted from NMR Suite professional software format (Chenomx Inc.) to Excel format (*csv) (Microsoft Corporation, Redmond, WA) for analysis. Orthogonal partial least squares to latent structures discriminant analysis (OPLS-DA) score plot of ^1^H NMR spectra was conducted using R to reveal the difference in metabolic patterns between the H and PT groups. Alpha diversity of the sample microbiota was estimated using the Shannon index. The Mann–Whitney U-test was used to compare significant differences of the alpha diversity indices between the different groups. To evaluate the similarity of microbial community structure among all samples, a principal coordinate analysis (PCoA) was performed. Analysis of similarities was calculated to compare the intra- and inter-group similarities based on the Bray Curtis distance, which was calculated by QIIME. A pre-trained Naive Bayes classifier, linked to the Human Oral Microbiome Database (eHOMD) 16S rRNA Extended RefSeq sequences (version 15.1) [19], was used to assign taxonomy to the unique representative sequences. Taxa that were significantly different among groups were identified by linear discriminant analysis effect size (LEfSe) with default settings [20]. Correlations between the relative abundance of microbes and metabolites were analyzed by sparse partial least squares (sPLS) regression analysis using the mixOmics package for R [21]. Within-group differences between pretreatment and follow-up were detected with the Wilcoxon test. Between-group differences were assessed with the Mann–Whitney test. Differences were considered significant at p < 0.05. All data were analyzed using Graph Pad Prism 7 software (GraphPad Software, Inc., San Diego, CA), R version 3.3.2 (R Foundation for Statistical Computing, Vienna, Austria), Cytoscape [22], and Excel (Microsoft Corporation).

### Data availability

The raw sequencing data have been deposited at NCBI GenBank under BioProject ID PRJNA625671 (BioSample SAMN14604593 – SAMN14604814). Please check the data using the below private reviewer link,

https://dataview.ncbi.nlm.nih.gov/object/PRJNA625671?reviewer=svrljq2nla5rfl69rdjgmqfusp

The codes are available at (http://doi.org/10.5281/zenodo.4438035).

## Results

### Patient characterization and subgrouping in the PT group

The demographic and clinical parameters of the participants are detailed in Table 1. PD, CAL, GI, and PI values were significantly higher in the PT group than in the H group. Based on metabolome clustering, the PT group was divided into two subgroups: PT_G1 and PT_G2. The PT_G2 subgroup had a significantly higher percentage of male patients and higher PI value than the PT_G1 subgroup.

**Table 1.** Epidemiologic characterization of patients

	Group				Significance			
	Healthy (n = 32)	Total CP (n = 79)	PT_G1 (n = 41)	PT_G2 (n = 38)	H vs total CP	H vs PT_G1	H vs PT_G2	PT_G1 vs PT_G2
Age (mean ± SD)	30.03 ± 7.19	53.99 ± 9.55	52.66 ± 8.49	54.26 ± 10.42	***	***	***	-
Gender (Male/Female)	15/18	48/31	19/22	29/9	-	-	-	**
Periodontitis Severity (Moderate/Severe)	-	46/33	26/15	20/18	-	-	-	-
PD (mean ± SD)	2.31 ± 0.29	3.52 ± 0.74	3.45 ± 0.81	3.60 ± 0.65	***	***	***	-
CAL (mean ± SD)	2.31 ± 0.29	3.91 ± 0.93	3.84 ± 0.89	3.99 ± 0.98	***	***	***	-
GI (mean ± SD)	0.12 ± 0.14	0.99 ± 0.49	0.99 ± 0.55	0.99 ± 0.45	***	***	***	-
PI % (mean ± SD)	19.96 ± 21.62	59.47 ± 25.67	53.65 ± 26.05	65.59 ± 24.10	***	***	***	*

* p < 0.05, ** p < 0.01, *** p < 0.001.

### NMR analysis of saliva

**Figure 1.**
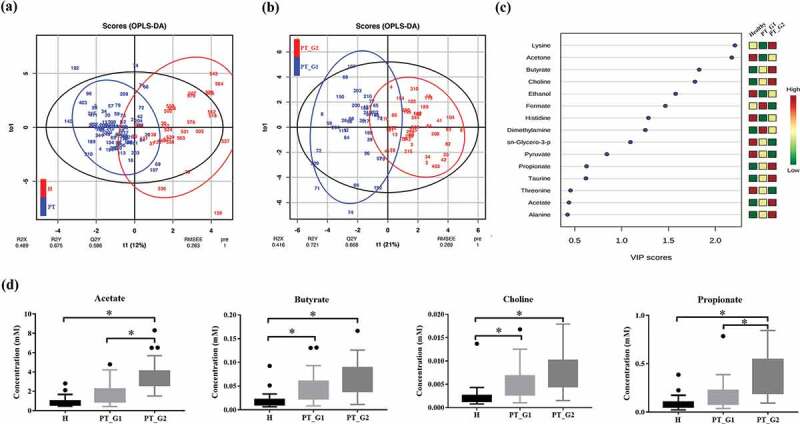
Saliva NMR data analysis. (a) OPLS/DA score plot of the spectra of saliva samples obtained from the healthy (H) and periodontitis (PT) groups. The saliva sample score plots of the H group were dispersed, while those of the PT group were clustered. (b) OPLS/DA score plot of PT patients. The patients were clustered into two subgroups (PT_G1 and PT_G2) (c) Variable Importance in Projection (VIP) scores of the important discriminatory metabolites obtained from the OPLS-DA models. (d) Metabolites identified to discriminate the H, PT_G1, and PT_G2 groups. * p < 0.05

A total of 28 metabolites was assigned in saliva. This was consistent with the findings of previous studies on saliva metabolites [23,24]. The normalized concentration of metabolites, including their average concentrations and standard deviation, is provided in Table S1. The OPLS-DA plot distinguished the H group from the PT group based on metabolite profiles (Figure 1(a)). While the H group metabolite profiles were scattered, suggesting heterogeneous composition, the metabolite profiles of the PT patients were clustered. When OPLS-DA was applied to the PT group, two clusters were found. Thus, the PT group was clustered into the PT_G1 and PT_G2 subgroups (Figure 1(b)) for further analyses.

To confirm the metabolic differences, Variable Importance in Projection (VIP) scores of the important discriminatory metabolites were obtained from the OPLS-DA models (Figure 1(c)). Compared with the H group, the butyrate and choline levels significantly increased in the PT_G1 patients. In the PT_G2 patients, the acetate, butyrate, choline, and propionate levels were significantly increased compared to those in the H group. The PT_G2 patients also showed higher concentration of acetate and propionate than PT_G1 patients (Figure 1(d)).

### Microbiome diversity and abundance

**Figure 2.**
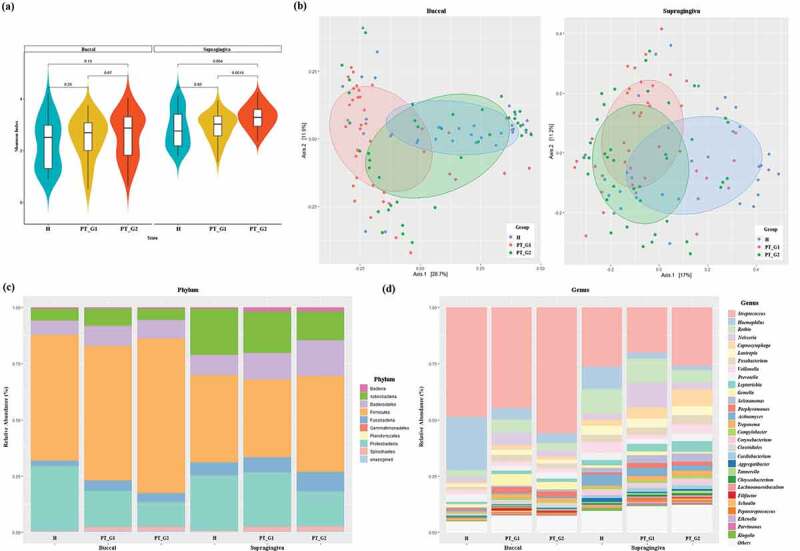
Bacterial community comparison. (a) Alpha diversity of the buccal and supragingival plaque samples calculated using the Shannon index. (b) Beta diversity of the buccal and supragingival plaque samples evaluated using principal coordinates analysis based on the abundance of species. (c) Relative abundance of bacterial phylum composition of buccal and supragingival plaques. (d) Relative abundance of bacterial genus composition of buccal and supragingival plaques

To characterize the microbiota composition, 16S rRNA V3-V4 targeted sequencing was performed. The diversity of the microbiota was estimated using the Shannon index (Figure 2(a)). Although not statistically significant, the Shannon index values for buccal and supragingival plaque samples were higher in the PT group than in the H group (Figure 2(a)). The PT_G2 patients displayed significantly higher diversity in supragingival plaque samples than the H and PT_G1 patients (p < 0.005). To analyze the distribution of microbiota, PCoA was conducted. This analysis revealed a pattern of clustering depending on the source groups of the buccal samples (Figure 2(b)). The PT_G2 and H buccal samples clustered together and could not be distinguished from each other, while the PT_G1 buccal samples clustered apart. Supragingival plaque samples were more dispersive, and the associated groups could not be distinguished from each other (Figure 2(b)). Thus, we could not find any distinctive feature in the diversity analysis.

Next, we analyzed the overall abundance at the phylum and genus levels. In buccal samples, the five most abundant phyla were *Firmicutes, Proteobacteria, Bacteroidetes, Actinobacteria*, and *Fusobacteria*, which represented more than 95% of the total phyla. In samples of supragingival plaque, the aforementioned five phyla also constituted the majority. In PT patients, the abundance of spirochetes was increased in both buccal and supragingival plaque samples (Figure 2(c)). At the genus level, among the 195 genera detected, the most abundant in buccal samples were *Streptococcus, Haemophilus, Rothia, Neisseria, Capnocytophaga, Lautropia, Fusobacterium, Veillonella*, and *Prevetella*. These constituted more than 75% of the total genera. In supragingival plaque samples, *Streptococcus, Haemophilus, Rothia, Neisseria, Prevotella, Fusobacterium, Actinomyces, Capnocytophaga, Veillonella*, and *Leptotrichia* represented more than 70% of the total genera (Figure 2(d)).

### Comparison of species taxa

**Figure 3.**
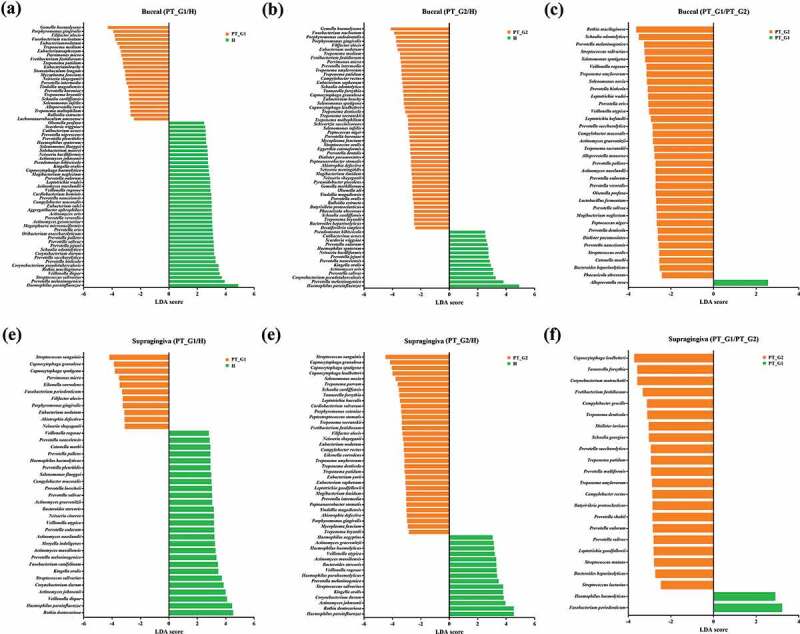
Comparison of significant taxa. LEfSe analysis was performed for all bacterial species, and the most discriminant ones (LDA score > 3.5) in decreasing order for each dataset are reported. (a) Buccal H vs. PT_G1, (b) Buccal H vs. PT_G2, (c) Buccal PT_G1 vs. PT_G2 (d) Supragingiva H vs. PT_G1, (e) Supragingiva H vs. PT_G2, and (f) Supragingiva PT_G1 vs. PT_G2

Next, the LEfSe algorithm [20] was used to determine discriminant bacterial species. In buccal samples, among the most significant taxa, *Gemella haemolysans, P. gingivalis, Filifactor alocis, Fusobacterium nucleatum*, and *T. forsythia* were more abundant in the PT group than in the H group. The abundance of *Haemophilus parainfluenzae, Prevotella melaninogenica*, and *Corynebacterium pseudotuberculosis* was significantly higher in the H group than in the PT group (Figure 3(a,b)). When the PT_G1 and PT_G2 subgroups were compared, significant taxa, including *T. denticola* and *Treponema socranskii*, were more abundant in the PT_G2 subgroup (Figure 3(c)). Concerning supragingival plaque samples, *Streptococcus sanguinis, Capnocytophaga* species (including *C. granulosa, C. sputigena*, and *C. leadbetteri), P. gingivalis, F. alocis*, and *T. socranskii* were significantly more abundant in the PT group than in the H group. In the H group, the abundance of *H. parainfluenzae, Rothia dentocariosa, Actinomyces johnsonii*, and *Kingella oralis* was significantly higher than that in the PT group (Figure 3(d,e)). When the PT_G1 and PT_G2 subgroups were compared, the abundance of *T. forsythia* and *Prevotella* species that included *P. salivae, P. oulurum, P. sacchrolytica*, and *P. loescheii* was significantly higher in the PT_G2 subgroup (Figure 3(f)). The relative abundance of the significant taxa determined by LEfSe was also plotted (Supplementary Figure S1).

### Integrated microbiome-metabolome network analysis

**Figure 4.**
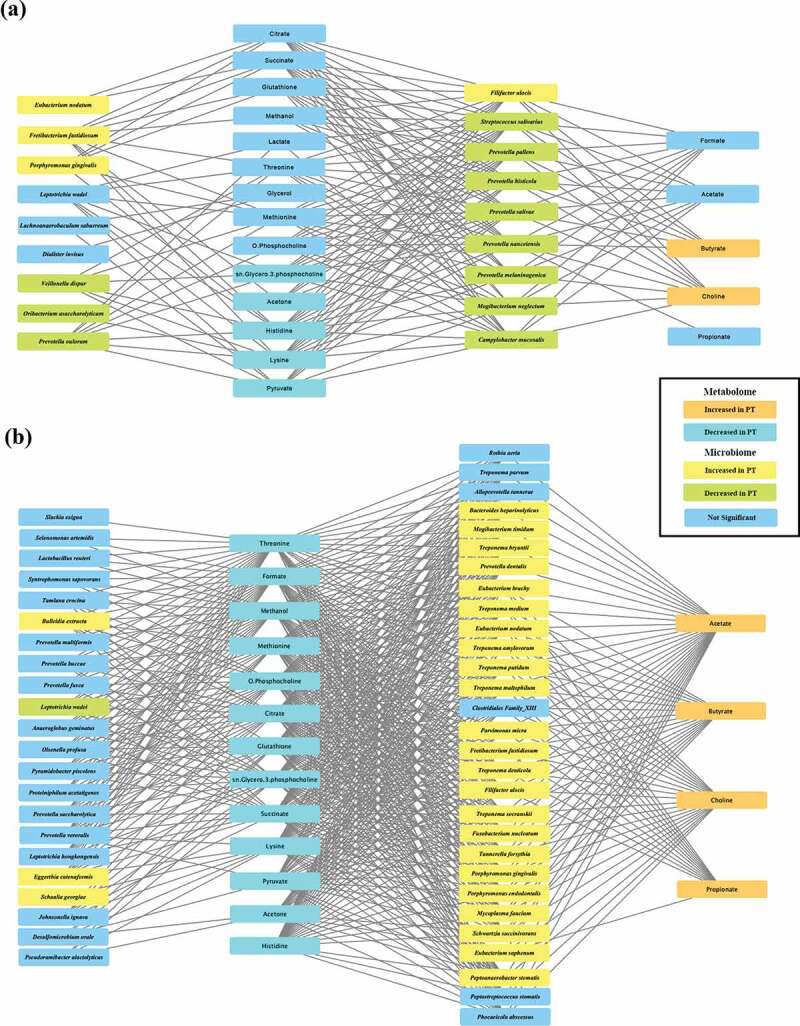
Integrated correlation-based network analysis of buccal microbiome and metabolites. Spares partial least squared correlation analysis was performed using mixOmics to determine the correlations between oral microbiome and identified metabolites. Yellow boxes denote bacterial species whose abundance was significantly increased in PT patients. Green boxes denote bacterial species whose abundance was significantly decreased in PT patients. Orange boxes denote metabolites whose concentrations were significantly increased in PT patients. Sky-blue boxes denote metabolites whose concentrations were significantly decreased in PT patients. (a) Network of the H group and PT_G1 subgroup. (b) Network of the H group and PT_G2 subgroup

To study the correlation between microbiome and metabolome, sPLS regression correlation analysis was performed using mixOmics. For visualization, bacterial species that correlated with short-chain fatty acids (SCFAs), whose concentrations were increased in the PT group, were selected as the first metabolite group. Bacterial species that were connected to the first metabolite group were considered the first microbial group. Metabolites whose concentrations decreased in the PT group were grouped as the second metabolite cluster. Bacterial species that correlated with only the second metabolite cluster were considered the second bacterial group. In the H and PT_G1 network, acetone, histidine, and lysine, whose concentrations were significantly decreased in the PT_G1 subgroup, showed comparable positive correlations with various bacterial species whose abundance was significantly decreased in the PT_G1 subgroup. These species included *Streptococcus salivarius, Prevotella. pallens, Prevotella histicola, P. salivae, Prevotella nanceiensis*, and *P. melaninogenica*. Among the bacterial species with increased abundance in the PT_G1 subgroup, only *F. alocis* abundance correlated with the butyrate and choline concentrations (Figure 4(a)). In the H and PT_G2 network, the concentrations of SCFAs, which included acetone, butyrate, choline, and propionate, displayed comparable correlations with bacterial species whose abundance was significantly increased in the PT_G2 subgroup. These species included *T. denticola, T. socranskii, F. alocis, T. forsythia, P. gingivalis, Porphyromonas endodontalis, Prevotella dentalis*, and *F. nucleatum*. Interestingly, the acetate concentration was correlated with the abundance of most bacteria, while the butyrate and choline concentrations showed a similar range of correlation with the abundance of classically classified periodontopathogens, including *T. denticola, F. alocis, T. socraskii, F. nucleatum, T. forsythia*, and *P. gingivalis*. The propionate concentration was correlated only with *T. denticola* and *F. alocis* abundance (Figure 4(b)).

### Clinical follow-up

In the follow-up, the PT_G1 and PT_G2 subgroups both displayed improved PD; no statistically significant differences were observed between the two groups at 3 to 6 months (p > 0.05, Table 2). In the PT_G1 subgroup, GI was also significantly improved, while PI was significantly improved in the PT_G2 subgroup. Between baseline and follow-up, PD reduction was 0.79 ± 0.0.58 mm and 1.26 ± 0.53 mm in the PT_G1 and PT_G2 subgroups, respectively. The reduction in CAL was 0.64 ± 0.83 mm and 0.87 ± 0.88 mm in the two subgroups, respectively. These differences were not statistically significant between the groups.

**Table 2.** Clinical parameters of baseline and follow-up evaluation in PT_G1 and PT_G2

	PT_G1 (n = 10)			PT_G2 (n = 7)		
	Baseline	Follow up	Change	Baseline	Follow up	Change
PD (mean ± SD)	3.32 ± 0.69	2.53 ± 0.35**	0.79 ± 0.58	3.61 ± 0.53	2.35 ± 0.53*	1.26 ± 0.53
CAL (mean ± SD)	3.58 ± 0.70	2.95 ± 0.41	0.64 ± 0.83	3.75 ± 0.53	2.88 ± 0.65	0.87 ± 0.88
GI (mean ± SD)	0.72 ± 0.53	0.20 ± 0.12*		1.06 ± 0.54	0.39 ± 0.35	
PI % (mean ± SD)	46.0 ± 15.9	24.5 ± 14.7		76.6 ± 23.0	32.5 ± 8.2*	

* baseline vs follow up: *p < 0.05, ** p < 0.01.

## Discussion

PT is the most prevalent type of periodontal disease. It affects approximately 70% of people aged 65 years and older. If untreated, PT leads to tooth loss. The risk of PT increases with age and is closely associated with systemic diseases, such as cardiovascular disease, diabetes, rheumatoid arthritis, and Alzheimer’s disease [25,26]. Therefore, early diagnosis of PT is important for both a healthy mouth and general health. However, currently, the diagnosis of PT is entirely based on clinical inspection and radiographic parameters [1,2]. Despite the knowledge that PT is a multifactorial chronic inflammatory disease caused by the interaction between oral pathogens and host tissues, no microbiological evaluation or assessment method for metabolites produced by periodontal pathogens is available for diagnosing this disease.

In this study, we applied multi-omics analysis of PT that combined microbiome analysis and metabolome analysis. Compared to single omics, multi-omics data analysis can provide researchers with a greater understanding of the disease [27,28]. First, we compared the metabolite profile of saliva samples collected from the H group with that of the samples collected from the PT group. While metabolite profiles of the H group were scattered, which suggest a heterogeneous composition, those from the PT group were more well clustered (Figure 1(a)). Among the metabolites identified, the butyrate and choline concentrations were significantly increased in the PT group compared to the H group. Butyrate has been suggested to contribute to the progression of periodontal disease [29–31]. The elevated concentrations of butyrate and propionate have been reported in gingival crevicular fluid collected from aggressive PT patients [32]. Thus, our findings are largely comparable to those of previous studies.

In the buccal microbiome analysis, *P. gingivalis, F. alocis, F. nucleatum*, and *T. forsythia* were among the significant species that were more abundant in the PT group (Figure 3(a,b)). In supragingival plaque samples, *Capnocytophaga* species, *Eikenella corrodens, P. gingivalis, F. alocis*, and *T. socranskii* were significantly more prevalent in the PT group (Figure 3(d,e)). Overall, our findings from the single microbiome analysis were largely comparable to those of previous studies [10,33–35].

Metabolites found in the saliva may originate from the microbiome on mucous membranes, such as the buccal mucosa or accumulated plaque. In single omics studies, since other parameters are not available, subgrouping is not commonly performed. Presently, we could cluster the PT patients depending on metabolite profiles and further characterize their microbiome composition. In the PT_G2 subgroup, which had a significantly higher concentration of short-chain fatty acids (including butyrate, acetate, choline and propionate), *Eubacterium nodatum, Dialister pneumosintes, Mogibacterium timidum, P. endodontalis, P. dentalis, T. denticola*, and *T. socranskii* were significantly more prevalent in buccal samples. In supragingival plaque samples collected from the PT_G2 group, the abundance of *Fretibacterium fastidiosum, Leptotrichia buccalis, M. timidum, Parvinoma micra, Peptostreptococcus stomatis, Selenomonas noxia, T. forsythia*, and *T. denticola* were significantly increased compared to that in the H group.

‘Refractory periodontitis’ was historically referred to as destructive periodontal diseases in patients who, when longitudinally monitored, demonstrated additional attachment loss at one or more sites, despite well-executed periodontal treatment and patient efforts to stop the progression of disease [36]. In 1993, the categories of PT associated with systemic disease and refractory PT were dropped from the classification [37]. As a consequence, only a limited number of studies have attempted to characterize the microbiome in ‘refractory’ PT. Interestingly, many bacteria that were more abundant in the PT_G2 subgroup were also in the list of bacteria found more commonly in ‘refractory’ PT than in general PT or healthy subjects. Bacterial species that include *P. micra, T. forsythia, Prevotella* spp., *P. gingivalis, E. nodatum, Eubacterium* spp., *Selenomonas* spp., and *Treponema* spp. are reportedly more prevalent in ‘refractory’ PT than general PT or the healthy state [38,39]. In more recent studies that utilized microarray analysis, subjects with ‘refractory’ PT displayed significantly higher frequencies of *P. gingivalis, P. endodontalis, F. alocis, E. corrodens, Eubacterium* spp. *E. nodatum, Selenomonas* spp., *S. noxia, T. forsythia, Prevotella* spp., *Treponema* spp., *Dialister invisus/pneumosintes, M. timidum, Peptostreptococcus spp*., and *P. micra* [11,40]. From a clinical perspective, we observed a significantly higher percentage of male patients in the PT_G2 subgroup. These patients displayed a higher PI than the PT_G1 subgroup (Table 1). The collective findings indicated that the oral microbiome of PT_G2 patients showed similar features to refractory PT patients who had responded poorly to conventional therapy in the past.

To study the interplay between microbiome and metabolome, a correlation analysis was performed using mixOmics [21]. In the H and PT_G2 network, the abundance of numerous periodontopathogens was positively correlated with the SCFA concentrations. Butyrate and choline showed a correlation with the classical periodontopathogens including *T. denticola, F. alocis, T. socraskii, F. nucleatum, T. forsythia*, and *P. gingivalis*. Butyrate and acetate are the major by-products of anaerobic metabolism in periodontopathogens, such as *Porphyromonas, Prevotella*, and *Fusobacterium* species [12,41]. *In vitro* culture studies have demonstrated that *P. gingivalis* and *F. nucleatum* produce more amounts of butyrate and acetate [31,42]. In gingival crevicular fluids obtained from patients with aggressive PT, the concentrations of acetic, propionic, and butyric acids were positively correlated with *P. gingivalis* and *T. denticola* abundance [43]. Thus, our network analysis was comparable to previous studies.

To determine the clinical outcome in the PT_G1 and PT_G2 subgroups, clinical parameters were checked in a follow-up visit, 3 to 6 months after periodontal treatment. Although there were no statistically significant differences, the PT_G2 group showed higher PD and CAL change. Considering the microbiome and its related metabolic profile, long-term follow-up should be performed to evaluate the clinical outcome.

In summary, we successfully integrated multi-omics data and identified a subgroup of periodontitis patients based on metabolomic and microbiome features. The strength of our study is that the use of the multi-omics approach provided rich and complementary understanding into the analysis of periodontitis compared to single-omics studies. Especially, interaction between the metabolites in the saliva and the related microbiomes in buccal sites support previous *in vivo* and *in vitro* studies [12,31,41,42]. Our study also shows the potential of multi-omics approach in predicting PT treatment outcome at the molecular level.

There are several limitations in this study. Our study uses a relatively small sample size suggesting that these findings should be validated in a larger clinical cohort. An adequate number of participants would be needed to allow stratification of participants into different sexes, clinical stage, or lifestyle. The lack of this stratification may influence the results of this study and should be evaluated in the future. Also, long-term clinical follow-up could be valuable. The findings open new avenues to explore the oral microbiome-metabolome associations for biomarker discovery for the diagnosis and management of PT.

## Supplementary Material

Supplemental Material
