# Supplementary material for: Molecular subgroup of periodontitis revealed by integrated analysis of the microbiome and metabolome in a cross-sectional observational study
Source: J Oral Microbiol. 2021 Mar 25;13(1):1902707. doi: 10.1080/20002297.2021.1902707 (PMC8008934; doi:10.1080/20002297.2021.1902707)
Supplement: Supplemental Material [file ZJOM_A_1902707_SM4030.zip › Supplementary files/Supplementary Figure Legendcleaan.docx]

**Supplementary Figure Legend**

**Figure S1.** Comparisons of microbiota that presented significantly different contents in both buccal (**A**) and supragingival plaque (**B**) samples of H, PT_G1 and PT_G2 groups. *p < 0.05.
