# Supplementary figures and images for: Molecular subgroup of periodontitis revealed by integrated analysis of the microbiome and metabolome in a cross-sectional observational study
Source: J Oral Microbiol. 2021 Mar 25;13(1):1902707. doi: 10.1080/20002297.2021.1902707 (PMC8008934; doi:10.1080/20002297.2021.1902707)

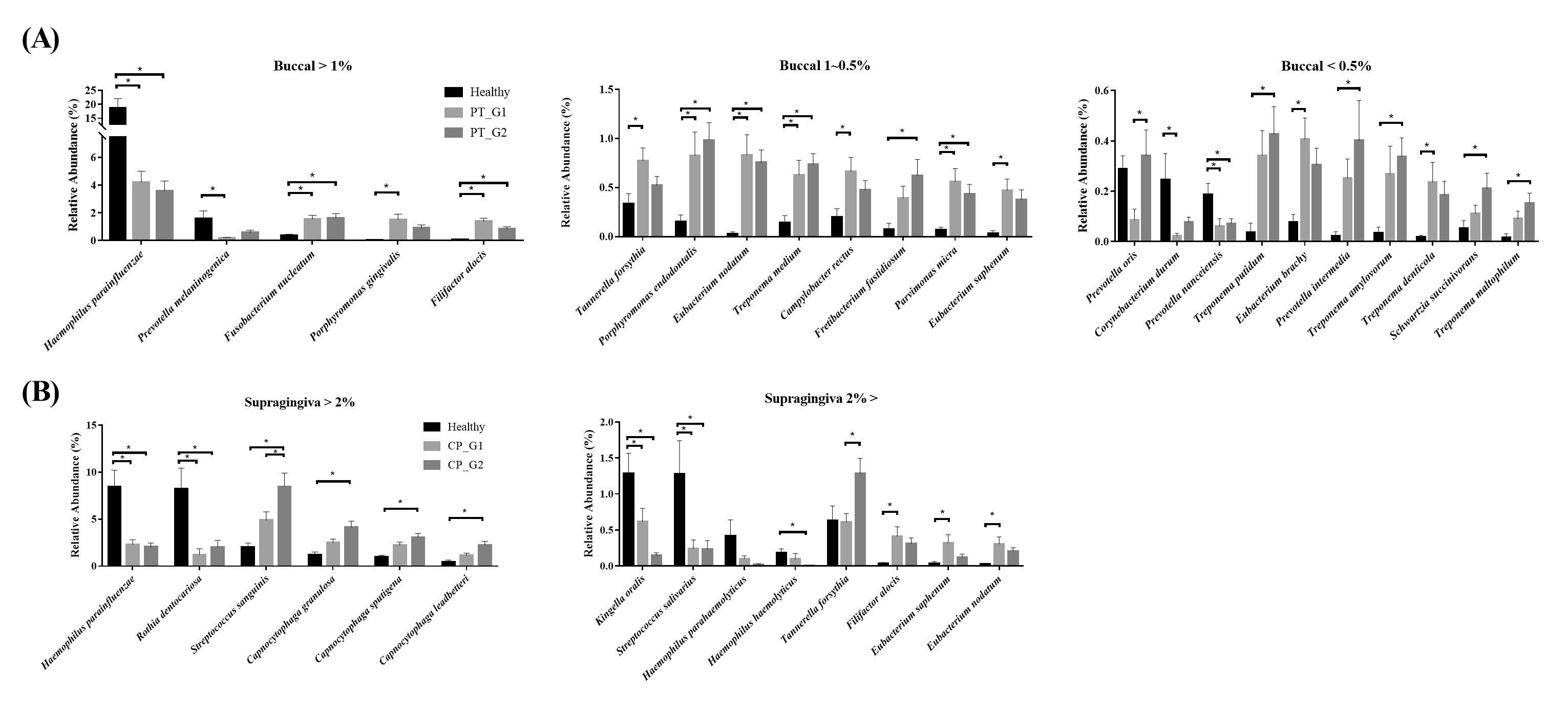

Supplement: Supplemental Material [file ZJOM_A_1902707_SM4030.zip › Supplementary files/Fig S1_Bon.tif]
